# In this month’s Bulletin

**DOI:** 10.2471/BLT.19.000419

**Published:** 2019-04-01

In the editorial section, M Claire Greene et al. (246) note a lack of attention to substance use disorders in refugee populations. Anayda Portela et al. (247) ask guideline developers to account for the complexity of health systems.

Andrey Shukshin (250–251) reports on Kazakhstan’s efforts to reform its health system four decades after the Declaration of Alma Ata. Mirfin Mpundu tells Gary Humphreys (252–253) about the challenges faced by African countries looking to contain antimicrobial resistance. 

## Burkina Faso

### A child’s right to an identity 

Evelina Martelli et al. (259–269) evaluate the impact of a programme designed to increase birth registration.

## Democratic Republic of the Congo, Ethiopia, United Republic of Tanzania

### Monitoring vital events 

Gustavo Corrêa et al. (306–308) argue for broader use of immunization data.

## India

### A decade’s progress

Suman Chakrabarti et al. (270–282) analyse equity and coverage of child services.

## Global

### Where there are no surgeons

Bhargava Mullapudi et al. (254–258) estimate gaps in access to pediatric surgery.

### Antimicrobial resistance surveillance

Cécile Aenishaenslin et al. (283–289) point out that better evidence is needed. 

### Documenting maternal and child health 

Keiko Osaki and Hirotsugu Aiga (296–305) argue for the adaption of home-based records.

### Unhealthy food and drink at sporting events

Robin Ireland et al. (290–296) call for scrutiny of sponsorship and advertising.

